# The effect of mental health conditions on the use of oral anticoagulation therapy in patients with atrial fibrillation: the FinACAF study

**DOI:** 10.1093/ehjqcco/qcab077

**Published:** 2021-10-22

**Authors:** Jussi Jaakkola, Konsta Teppo, Fausto Biancari, Olli Halminen, Jukka Putaala, Pirjo Mustonen, Jari Haukka, Miika Linna, Janne Kinnunen, Paula Tiili, Aapo L Aro, Juha Hartikainen, K E Juhani Airaksinen, Mika Lehto

**Affiliations:** Heart Center, Turku University Hospital, and University of Turku, Hämeentie 11, Turku, Finland; Heart Unit, Satakunta Central Hospital, Sairaalantie 3, FI-28500 Pori, Finland; Heart Center, Turku University Hospital, and University of Turku, Hämeentie 11, Turku, Finland; Heart and Lung Center, Helsinki University Hospital, and University of Helsinki, Haartmaninkatu 4, Helsinki, Finland; Research Unit of Surgery, Anesthesia and Critical Care, University of Oulu, Kajaanintie 50, Oulu, Finland; Clinica Montevergine, GVM Care & Research, Via Mario Malzoni 5, Mercogliano, Italy; Department of Industrial Engineering and Management, Aalto University, Espoo, Finland; Department of Neurology, Helsinki University Hospital, and University of Helsinki, Haartmaninkatu 4, Helsinki, Finland; Heart Center, Turku University Hospital, and University of Turku, Hämeentie 11, Turku, Finland; Department of Population Health, University of Helsinki, Yliopistonkatu 4, Helsinki, Finland; Department of Neurology, Helsinki University Hospital, and University of Helsinki, Haartmaninkatu 4, Helsinki, Finland; Department of Health and Social Management, University of Eastern Finland, Yliopistonranta 1, Kuopio, Finland; Department of Neurology, Helsinki University Hospital, and University of Helsinki, Haartmaninkatu 4, Helsinki, Finland; Department of Neurology, Helsinki University Hospital, and University of Helsinki, Haartmaninkatu 4, Helsinki, Finland; Heart and Lung Center, Helsinki University Hospital, and University of Helsinki, Haartmaninkatu 4, Helsinki, Finland; Heart Center, Kuopio University Hospital, Puijonlaaksontie 2, Kuopio, Finland; Heart Center, Turku University Hospital, and University of Turku, Hämeentie 11, Turku, Finland; Heart and Lung Center, Helsinki University Hospital, and University of Helsinki, Haartmaninkatu 4, Helsinki, Finland; Department of Internal Medicine, Lohja Hospital, Sairaalatie 8, Lohja, Finland

**Keywords:** Atrial fibrillation, Anticoagulation, Mental health, Depression, Inequality

## Abstract

**Aims:**

Little is known about the effects of mental health conditions (MHCs) on the utilization of oral anticoagulation (OAC) therapy in atrial fibrillation (AF) patients. We aimed to assess whether MHCs affect initiation of OAC therapy among AF patients with special focus on non-vitamin K antagonist oral anticoagulants (NOACs).

**Methods and results:**

The Finnish AntiCoagulation in Atrial Fibrillation (FinACAF) registry included all 239 222 patients diagnosed with incident AF during 2007–18 in Finland identified from national registries covering primary to tertiary care and drug purchases. Patients with previous depression, bipolar disorder, anxiety disorder, or schizophrenia diagnosis or a fulfilled psychiatric medication prescription within the year preceding the AF diagnosis were classified to have any MHC. The main outcome was OAC initiation, defined as first fulfilled OAC prescription after AF diagnosis. The patients’ mean age was 72.7 years and 49.8% were female. The prevalence of any MHC was 19.9%. A lower proportion of patients with any MHC compared with those without MHCs were initiated on OAC therapy (64.9% vs. 73.3%, *P* < 0.001). Any MHC was associated with lower incidence of OAC initiation [adjusted subdistribution hazard ratio (aSHR) 0.867; 95% confidence interval (CI) 0.856–0.880], as were depression (aSHR 0.868; 95% CI 0.856–0.880), bipolar disorder (aSHR 0.838; 95% CI 0.824–0.852), anxiety disorder (aSHR 0.840; 95% CI 0.827–0.854), and schizophrenia (aSHR 0.838; 95% CI 0.824–0.851), during the entire follow-up. Any MHC remained associated with impaired incidence of OAC initiation also in the NOAC era during 2015–18 (aSHR 0.821; 95% CI 0.805–0.837).

**Conclusion:**

MHCs are common among AF patients, and they are associated with a lower rate of OAC initiation even during the NOAC era.

## Introduction

Atrial fibrillation (AF) is a common arrhythmia that affects up to 3% of the general population and is an important risk factor of ischaemic stroke observed in over a third of all stroke patients.^1^ Fortunately, stroke risk may be effectively reduced with oral anticoagulation (OAC) therapy, with either vitamin K antagonists (VKAs), which require frequent dose monitoring, or the newer generation non-vitamin K antagonist oral anticoagulants (NOACs), with which dose monitoring is not necessary.^2^ Comorbid mental health conditions (MHCs) are exceedingly common among AF patients, with the prevalence ranging from 18% to 38% across previous reports.^3,4^

Patients with MHCs have a high prevalence of cardiovascular risk factors and cardiovascular disease and experience increased cardiovascular mortality.^5–9^ Life expectancy among patients with severe MHCs is reduced by as much as 15–20 years, mainly due to somatic causes, with cardiovascular disease being among the most important causes behind lost life-years.^5,10–12^ Several studies have demonstrated that patients afflicted by MHCs are frequently undertreated for unrelated medical conditions.^13,14^ However, data on the effects of MHCs on the utilization of OAC therapy in patients with AF are currently limited. A lower prevalence of OAC therapy among patients with MHCs has been reported in a few prior studies, although these data are derived mostly from the first decade of the 2000s, thus pertaining mainly to VKA therapy, and they largely lack a primary care perspective.^4,15–17^ Specifically, whether the subsequent emergence of the easier-to-use NOACs has improved OAC coverage among this group of patients is poorly understood. The aim of this study was to determine, by utilizing a nationwide cohort covering all patients diagnosed with AF during 2007–18 in Finland at all levels of care, whether the presence of MHCs affects the rate of initiation of OAC therapy in AF patients and whether the introduction of NOACs has improved OAC coverage among patients with AF afflicted by MHCs.

## Methods

### Study population

The Finnish AntiCoagulation in Atrial Fibrillation (FinACAF) study (ClinicalTrials identifier: NCT04645537; ENCePP identifier: EUPAS29845) is a retrospective nationwide registry-based cohort study comprising all patients recorded with a diagnosis of AF during 2004–18 in Finland. Patients were identified from three national healthcare registers [hospitalizations and outpatient specialist visits: Care Register for Health Care (HILMO); primary healthcare: Register of Primary Health Care Visits (AvoHILMO); and the National Reimbursement Register upheld by the Social Insurance Institute (KELA)].

**Figure 1:**
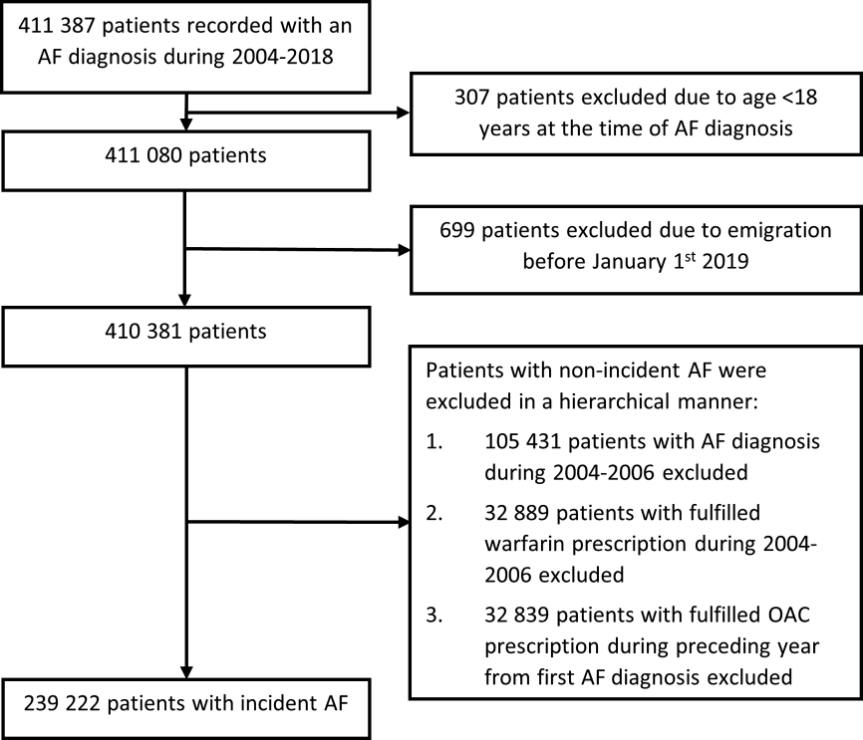
Flow chart of the study patient selection process. AF, atrial fibrillation.

The inclusion criteria for the registry were an ICD-10 (International Classification of Diseases, 10th revision) diagnosis code I48 for AF recorded at any time during 2004–18 in any of the aforementioned registries. Altogether, 411 387 patients with AF were identified. The exclusion criteria were age <18 years on index date and permanent migration abroad before 1 January 2019. The current substudy focused only on those patients who had incident AF, i.e. were diagnosed with AF for the first time during the study period. Thus, patients with a recorded AF diagnosis or fulfilled warfarin prescription during 2004–06 were excluded, as these patients had little recorded medical history and most likely many had a prior diagnosis of AF before the study inception. Additionally, patients with a fulfilled warfarin or NOAC prescription within the preceding year from the index date were excluded as most of them likely had a prior diagnosis of AF. Finally, there were 239 222 patients included in the current analysis (*Figure 1*).

**Figure 2:**
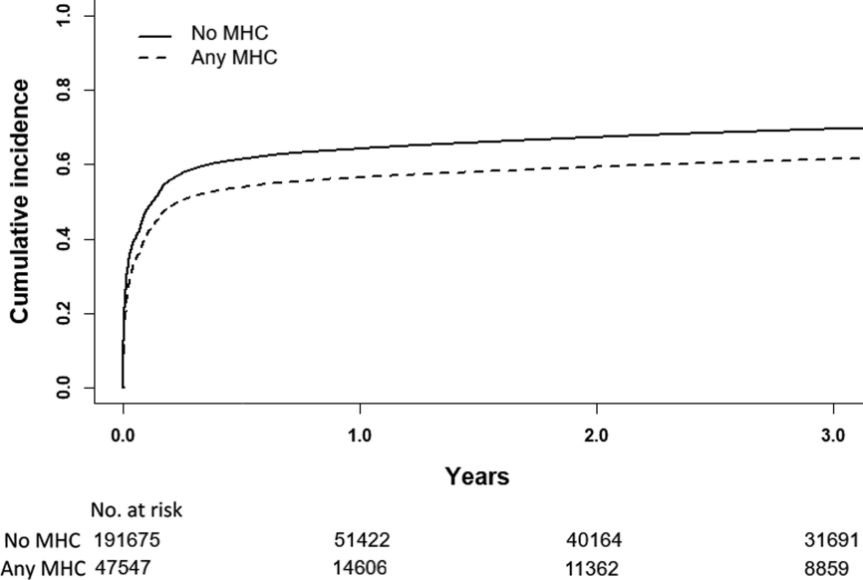
Cumulative incidence curve of oral anticoagulation initiation in patients with and without mental health conditions. MHC, mental health condition.

### Study protocol

During the study period, cohort entry occurred on the date of the first recorded AF diagnosis. Follow-up ended on the date of death or 31 December 2018, whichever occurred first. The primary outcome measure of the current study was the initiation of OAC therapy. OAC initiation was considered to occur on the date of first fulfilled OAC prescription after the cohort entry.

The MHCs of interest were any MHC, depression, bipolar disorder, anxiety disorder, and schizophrenia. Patients were classified into these groups if they were recorded with the ICD-10 diagnosis code or International Classification of Primary Care, second edition (ICPC-2) entry of the specific condition prior to first AF diagnosis [depression (ICD-10: F32, F33, and F34.1; ICPC-2: P76), anxiety disorder (ICD-10: F40–F42 and F43.1; ICPC-2: P74), bipolar disorder (ICD-10: F31; ICPC-2: P73), and schizophrenia (ICD-10: F20; ICPC-2: P72)]. Patients with more than one of these conditions were classified to each diagnostic category separately. Patients were classified to have any MHC if they had one or more of these specific MHCs or had fulfilled a prescription of an antipsychotic, antidepressant, anxiolytic, or mood-stabilizing medication within the year preceding the first AF diagnosis [Anatomic Therapeutic Chemical (ATC) codes: N05A, N05BE01, and N06A]. Medication data were not utilized to further classify patients in the specific MHC groups as many psychiatric drugs are commonly utilized for various differing conditions and it is not possible to determine the exact indication of each prescription.

Diagnoses of comorbidities were constructed in a hierarchal manner. If a patient had an ICD-10 diagnosis or ICPC-2 entry of the comorbidity recorded at any time before cohort entry in any of the included national registries, they were considered to have the condition. Additionally, if a patient was entitled to reimbursement for the medication for the comorbidity or had fulfilled a prescription for the condition within the year preceding AF diagnosis, they were also considered to have the comorbidity. The definitions of comorbidities are detailed in the Supplementary material online, *Table S1*.

### Study ethics

The study protocol was approved by the Ethics Committee of the Medical Faculty of Helsinki University, Helsinki, Finland (15/2017), and research permission was granted by the Helsinki University Hospital (HUS/46/2018). Respective permissions were obtained from KELA (138/522/2018), the Finnish Institute for Health and Welfare (THL) (THL/2101/5.05.00/2018), and the Population Register Center (VRK/1291/2019-3). Informed consent was waived due to the retrospective-registry nature of the study. The study conforms to the Declaration of Helsinki as revised in 2002.

### Statistical analysis

Statistical analyses were performed with the IBM SPSS Statistics software (version 27.0, SPSS Inc., Chicago, IL), R (version 4.0.5, https://www.R-project.org) and Stata (version 15.1, StataCorp LLC, TX, USA). The normality assumption of continuous variables was assessed visually and with the Kolmogorov–Smirnov test of normality. Continuous data are presented as mean (standard deviation) or median [interquartile range] and categorical variables as absolute number and percentage. Fisher's exact test was used to compare differences between proportions, and the Mann–Whitney *U* test and independent samples *t*-test to analyse continuous variables. Poisson regression analyses were conducted to determine the incidence rate ratios (IRRs) for OAC initiation in the different MHC groups. Initiation of OACs may be hindered by death occurring during the follow-up. Therefore, Fine–Gray subdistribution hazard competing risk analyses were performed to estimate the effect of MHCs on the cumulative incidence of initiation of OAC therapy considering all-cause mortality as a competing event. A sensitivity analysis was also performed evaluating only patients entering the study cohort during the years 2015–18. Risk estimates from the Fine–Gray models are summarized as subdistribution hazard ratios (SHRs) with 95% confidence intervals (CIs). In the Poisson and Fine–Gray models, adjustments were made for the following baseline covariates: age, gender, hypertension, dyslipidaemia, heart failure, diabetes, prior stroke, vascular disease, renal failure or dialysis, liver cirrhosis or failure, alcohol abuse, prior bleeding episodes, and dementia as well as year of cohort entry in the Poisson regression analyses.

The study cohorts differed in several baseline characteristics and therefore an additional propensity score matching analysis was performed to avoid bias in comparative analysis. A propensity score was calculated using a non-parsimonious logistic regression with any MHC as the dependent variable including the following variables into the regression model: age, gender, hypertension, dyslipidaemia, heart failure, diabetes, prior stroke, vascular disease, renal failure or dialysis, liver cirrhosis or failure, alcohol abuse, prior bleeding episodes, dementia, CHA_2_DS_2_-VASc [Congestive Heart Failure, Hypertension, Age ≥ 75 Years, Diabetes, History of Stroke, Vascular Disease, Age 65–74 Years, Sex Category (Female)] score, and HAS-BLED (Hypertension, Abnormal Renal Function or Liver Enzymes, Prior Stroke, Bleeding History, Labile INR, Elderly, Drugs or Alcohol) score. One-to-one matching was performed with the nearest neighbour approach using a calliper width of 0.1, i.e. 0.2 of the standard deviation of the logit (0.55). Balance between the study cohorts was evaluated by estimating standardized differences. Standardized difference <0.10 was considered an acceptable balance between the study cohorts. Subsequently, a Fine–Gray subdistribution hazard competing risk analysis was performed to estimate the cumulative incidence of OAC initiation in propensity score matched pairs with and without any MHC.

## Results

The mean age of the patients was 72.7 (standard deviation 13.2) years, and 119 046 (49.8%) of them were female. Altogether, 90.1% of the men and 96.1% of the women had CHA_2_DS_2_-VASc scores ≥1 and ≥2, respectively, and were therefore eligible for OAC therapy. The overall prevalence of any MHC at cohort entry was 19.9% (47 547 patients). Patients with any MHC were more often female, had generally more cardiovascular risk factors, and suffered more often from vascular disease compared with those with no history of MHC (*Table 1*).

**Table 1:** The baseline characteristics and medications of the study patients

	No MHC (*n* = 191 675)	Any MHC (*n* = 47 547)	Depression (*n* = 10 920)	Bipolar disorder (*n* = 1129)	Anxiety disorder (*n* = 4382)	Schizophrenia (*n* = 1560)
Baseline characteristics						
Age, years (SD)	72.6 (13.0)	72.8 (14.2)	69.8 (14.4)	64.2 (13.0)	66.2 (16.3)	69.6 (11.7)
Female sex	90 754 (47.3)	28 292 (59.5)	6494 (59.5)	523 (46.3)	2675 (61.0)	823 (52.8)
Hypertension	147 803 (77.1)	38 637 (81.3)	8944 (81.9)	892 (79.0)	3570 (81.5)	1079 (69.2)
Dyslipidaemia	91 867 (47.9)	23 854 (50.2)	5688 (52.1)	574 (50.8)	2136 (48.7)	600 (38.5)
History of heart failure	31 886 (16.6)	9810 (20.6)	2050 (18.8)	186 (16.5)	702 (16.0)	471 (30.2)
Diabetes	40 143 (20.9)	11 733 (24.7)	2962 (27.1)	360 (31.9)	1005 (22.9)	559 (35.8)
Previous stroke	27 463 (14.3)	8605 (18.1)	1938 (17.7)	196 (17.4)	693 (15.8)	231 (14.8)
Vascular disease^a^	48 815 (25.5)	13 598 (28.6)	3057 (28.0)	241 (21.3)	1058 (24.1)	341 (21.9)
Renal failure or dialysis	3816 (2.0)	1197 (2.5)	319 (2.9)	32 (2.8)	117 (2.7)	34 (2.2)
Liver cirrhosis or failure	911 (0.5)	383 (0.8)	132 (1.2)	16 (1.4)	52 (1.2)	10 (0.6)
Alcohol abuse	5081 (2.7)	4354 (9.2)	1856 (17.0)	311 (27.5)	746 (17.0)	153 (9.8)
Bleeding history	20 625 (10.8)	7 055 (14.8)	1874 (17.2)	185 (16.4)	740 (16.9)	228 (14.6)
Dementia	10 576 (5.5)	7335 (15.4)	1480 (13.6)	91 (8.1)	416 (9.5)	187 (12.0)
CHA_2_DS_2_-VASc score	3 [2–5]	4 [2–5]	4 [2–5]	3 [2–4]	3 [2–5]	3 [2–5]
0	10 097 (5.3)	1796 (3.8)	469 (4.3)	75 (6.6)	229 (5.2)	62 (4.0)
1	21 137 (11.0)	4469 (9.4)	1205 (11.0)	189 (16.7)	659 (15.0)	169 (10.8)
≥2	160 441 (83.7)	41 282 (86.8)	9246 (84.7)	865 (76.6)	3494 (79.7)	1329 (85.2)
HAS-BLED score^b^	2 [1–2]	2 [1–3]	2 [1–3]	2 [1–3]	2 [1–3]	2 [1–2]
0	14 907 (7.8)	2560 (5.4)	594 (5.4)	79 (7.0)	317 (7.2)	106 (6.8)
1	45 613 (23.8)	9985 (21.0)	2467 (22.6)	295 (26.1)	1118 (25.5)	485 (31.1)
2	87 750 (45.8)	20 578 (43.3)	4376 (40.1)	409 (36.2)	1675 (38.2)	601 (38.5)
≥3	43 405 (22.6)	14 424 (30.3)	3483 (31.9)	346 (30.6)	1272 (29.0)	368 (23.6)
Medications						
ADP inhibitors	8771 (4.6)	2682 (5.6)	610 (5.6)	57 (5.0)	250 (5.7)	53 (3.4)
Dipyridamole	8968 (4.7)	3193 (6.7)	597 (5.5)	61 (5.4)	193 (4.4)	68 (4.4)
Beta blockers	94 377 (49.2)	26 005 (54.7)	5815 (53.3)	575 (50.9)	2354 (53.7)	695 (44.6)
ACE blockers or AT inhibitors	89 601 (46.7)	22 690 (47.5)	5123 (46.9)	485 (43.0)	1951 (44.5)	602 (38.6)
DHP calcium channel blockers	50 983 (26.6)	13 284 (27.9)	3018 (27.6)	277 (24.5)	1176 (26.8)	345 (22.1)
Diuretics	67 031 (35.0)	19 387 (40.8)	4050 (37.1)	386 (34.2)	1401 (32.0)	569 (36.5)
Statins	68 377 (35.7)	17 432 (36.7)	3873 (35.5)	410 (36.3)	1434 (32.7)	448 (28.7)
Insulin	10 320 (5.4)	3504 (7.4)	896 (8.2)	111 (9.8)	257 (5.9)	168 (10.8)
Oral diabetes medications	31 064 (16.2)	9179 (19.3)	2325 (21.3)	283 (25.1)	763 (17.4)	460 (29.5)
Antidepressants	0 (0.0)	29 554 (62.2)	6577 (60.2)	502 (44.5)	2419 (55.2)	417 (26.7)
Antipsychotics	0 (0.0)	12 712 (26.7)	3245 (29.7)	837 (74.1)	1424 (32.5)	1373 (88.0)

Values are presented as absolute number (percentage), mean (standard deviation), or median [interquartile range].

ACE, angiotensin-converting enzyme; AT, angiotensin; CHA_2_DS_2_-VASc, Congestive Heart Failure, Hypertension, Age ≥ 75 Years, Diabetes, History of Stroke, Vascular Disease, Age 65–74 Years, Sex Category (Female); DHP, dihydropyridine; HAS-BLED, Hypertension, Abnormal Renal Function or Liver Enzymes, Prior Stroke, Bleeding History, Labile INR, Elderly, Drugs or Alcohol; INR, international normalized ratio; MHC, mental health condition; SD, standard deviation.

a Coronary artery disease or peripheral vascular disease.

b Modified HAS-BLED score without labile INR.

A lower overall proportion of OAC initiation was observed in patients with any MHC than in those without MHC [30 853 (64.9%) vs. 140 417 (73.3%) patients, *P* < 0.001] during the entire follow-up. Among patients with any MHC, altogether 38.8% had initiated OAC therapy at 1 month of follow-up, 53.6% at 6 months of follow-up, 56.1% at 1 year of-follow-up, and 60.5% at 3 years of follow-up. Among those patients with no MHC, these figures were 45.3%, 61.0%, 63.7%, and 68.5%, respectively. In patients who did start OAC therapy, median time to therapy initiation was 16 [2–75] days in patients with any MHC as compared with 12 [1–67] days in those with no MHC (*P* < 0.001).

In unadjusted analyses as well as after adjusting for confounding factors, any MHC was associated with a lower rate of OAC initiation compared with no MHC both in the Poisson regression model and in the Fine–Gray subdistribution hazard competing risks model (*Figure 2* and *Tables 2* and *3*). Similar associations with lower OAC initiation were observed individually regarding depression, bipolar disease, anxiety, and schizophrenia in comparison with patients without these conditions in both the unadjusted and adjusted Poisson and Fine–Gray models (*Tables 2* and *3*).

**Table 2:** Incidence of oral anticoagulation initiation according to the presence of mental health conditions

Clinical condition	Events	Proportion of patients with events (%)	Patient-years	Incidence (per patient-year)	Unadjusted IRR	Adjusted IRR
No MHC	140 417	73.3	253 026.88	0.555 (0.552-0.558)	(Reference)	(Reference)
Any MHC	30 853	64.9	70 108.59	0.440 (0.435–0.445)	0.793 (0.783–0.803)	0.827 (0.817–0.838)
Depression	7170	65.7	14 444.54	0.496 (0.485–0.508)	0.894 (0.873–0.916)	0.852 (0.831–0.873)
Bipolar disorder	727	64.4	1780.45	0.408 (0.380–0.439)	0.736 (0.684–0.791)	0.825 (0.767–0.888)
Anxiety disorder	2751	62.8	6413.79	0.429 (0.413–0.445)	0.773 (0.744–0.803)	0.789 (0.760–0.820)
Schizophrenia	945	60.6	1934.25	0.489 (0.458–0.521)	0.880 (0.826–0.939)	0.838 (0.786–0.893)

Adjusted IRRs estimated by Poisson regression.

95% confidence intervals in parentheses.

IRR, incidence rate ratio; MHC, mental health condition.

**Table 3:** Three-year cumulative incidences and adjusted risk estimates of initiation of oral anticoagulation therapy in patients with and without mental health conditions

Clinical condition	MHC (%)	No MHC (%)	Unadjusted SHR, 95% CI	Adjusted SHR, 95% CI^a^
Any MHC	61.4 (61.0–61.9)	69.4 (69.2–69.6)	0.807 (0.797–0.817)	0.867 (0.856–0.880)
Depression	63.8 (62.9–64.7)	69.4 (69.2–69.6)	0.844 (0.824–0.864)	0.868 (0.856–0.880)
Bipolar disorder	61.1 (59.6–62.6)	69.4 (69.2–69.6)	0.796 (0.766–0.827)	0.838 (0.824–0.852)
Anxiety disorder	61.2 (58.2–64.0)	69.4 (69.2–69.6)	0.793 (0.736–0.853)	0.840 (0.827–0.854)
Schizophrenia	59.1 (56.6–61.6)	69.4 (69.2–69.6)	0.746 (0.701–0.795)	0.838 (0.824–0.851)

CI, confidence interval; MHC, mental health condition; SHR, subdistribution hazard ratio.

a Values are cumulative incidences with 95% confidence intervals in parentheses. Risk estimates were adjusted for age, gender, hypertension, dyslipidaemia, heart failure, diabetes, prior stroke or transient ischaemic ischaemia, vascular disease, renal failure or dialysis, liver cirrhosis or failure, alcohol abuse, prior bleeding episodes and dementia.

Propensity score matching resulted in 47 095 pairs with similar characteristics, as demonstrated by standardized differences less than 0.10 in all baseline covariates (Supplementary material online, *Table S2*). Among the propensity score matched pairs, Fine–Gray subdistribution hazard competing risk analysis demonstrated that any MHC was associated with lower initiation of OAC compared with patients without MHC (cumulative incidence at 3 years: 57.7% vs. 62.2%, SHR 0.892; 95% CI 0.877–0.907). Among propensity score matched pairs with CHA_2_DS_2_-VASc score ≥2, at 3 years the cumulative incidence of initiation of OAC was 61.1% among patients with any MHC and 66.3% among patients without MHC (SHR 0.880; 95% CI 0.865–0.896).

**Figure 3:**
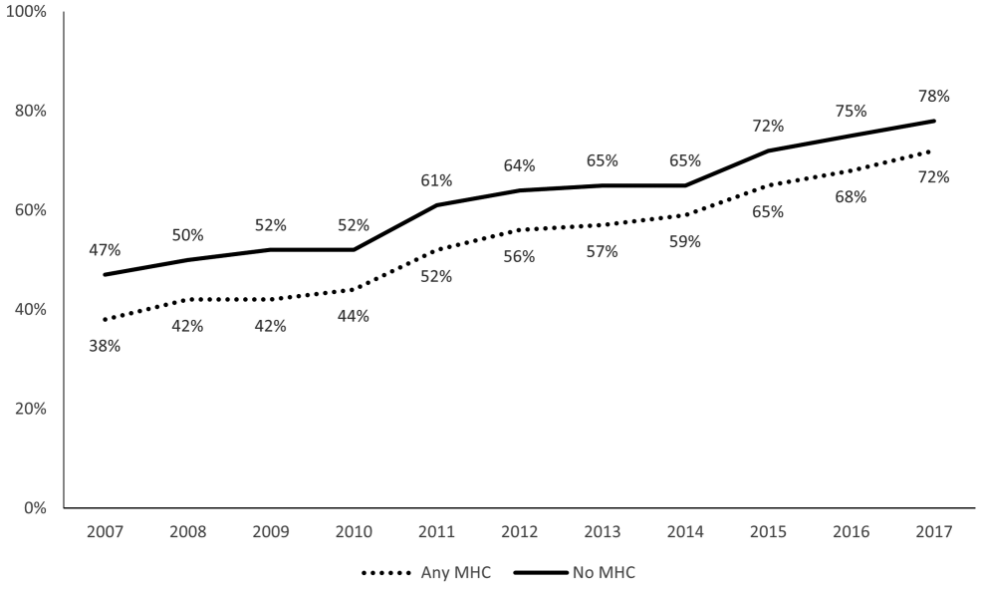
Proportion of patients initiated with oral anticoagulation by 1 year of follow-up according to year of atrial fibrillation diagnosis. MHC, mental health condition.

A continuous increase in OAC initiation was observed during the study period. At 1 year of follow-up, the rate of OAC initiation improved from 45.3% in patients diagnosed with AF during 2007 to 76.8% in those diagnosed during 2017. Although the overall OAC coverage significantly improved during the study period, patients with any MHC presented consistently with a lower rate of OAC initiation compared with patients with no MHC (*Figure 3*). Among patients entering the cohort in the NOAC era after 2014 (*n* = 89 284), the rate of OAC initiation was 70.2% in those with any MHC compared with 76.8% in those without. Fine–Gray subdistribution hazard competing risk analysis of patients entering the cohort after 2014 showed that any MHC remained associated with lower cumulative incidence of OAC initiation also during the NOAC era [adjusted SHR 0.908 (95% CI 0.888–0.927]. Fine–Gray analyses were also conducted separately according to the year of AF diagnosis and any MHC remained associated with reduced OAC initiation during each year of the study period (Supplementary material online, *Table S3*).

During the entire follow-up, OAC therapy was initiated with an NOAC in 9092 (29.5%) patients with any MHC and 43 989 (31.3%) patients without MHC among those patients who did start OAC treatment. The share of NOAC initiation increased greatly during the follow-up in both patients with and those without MHCs. Among the patients who started OAC therapy during 2018 (*n* = 21 659), treatment was initiated with an NOAC in 3602 (89.8%) patients with any MHC and 16 309 (92.4%) patients with no MHC.

## Discussion

The current study documented that MHCs are common among patients with newly diagnosed AF, with a prevalence of approximately 20%. Importantly, MHCs, including depression, bipolar disorder, anxiety disorder, and schizophrenia, were associated with impaired OAC initiation in comparison with patients without these conditions even after adjusting for baseline covariates and important bleeding risk factors. Although overall significant improvement was noted in OAC coverage during the study period, patients with MHCs presented consistently with lower rates of OAC initiation compared with patients without these conditions. Importantly, MHCs retained their association with poor OAC initiation also during the NOAC era.

Previous research on OAC initiation in AF patients affected by MHCs has limitations. Lower prevalence of warfarin therapy in patients with MHCs in comparison with those without psychiatric comorbidity has been reported in a few prior cohort studies of mostly small sample sizes.^4,15,16^ The main shortcoming is that these data are derived mainly from the first decade of the 2000s and there is paucity of information on whether the improved understanding of the significance of AF as a stroke risk factor and the emergence of the fixed-dose NOACs have improved OAC coverage among patients with MHCs in the recent years. To date, this issue has been addressed only by Fenger-Grøn *et al*. in a single cohort of over 147 000 Danish patients diagnosed with incident AF during 2005–16 in a hospital setting, although relatively few patients receiving NOACs were included.^17,18^ They observed that depression (adjusted proportion difference −6.6%), bipolar disorder (adjusted proportion difference −12.7%), and schizophrenia (adjusted proportion difference −24.5%) were associated with decreased OAC initiation, although the difference tended to attenuate after the introduction of NOACs in patients with depression and bipolar disorder.^17,18^ These findings are partly in contrast to the current study, wherein no material improvement in relative OAC deficit in patients with MHCs compared with those without MHCs was observed after entering the NOAC era. This may at least in part be related to differing definitions of MHCs utilized between the studies. Of note, the Danish investigators lacked primary care data, which limits the generalizability of their results in relation to our work, which included patients from all levels of care. Differences in local practices of care may also contribute to the differing results between these studies, although the healthcare systems and culture are quite similar in Denmark and Finland.

The observed lower rate of OAC initiation in patients with MHCs is most likely multifactorial. It has been reported that contraindications for OAC medications are more prevalent in patients with MHCs than in those not afflicted by these conditions.^15^ A particular concern in this regard may be frequently excessive alcohol consumption among patients with MHCs, which has been linked with poor anticoagulation control, increased risks of falls and physical injury, and major bleeding.^19–23^ However, in a small cohort study, Walker *et al.* observed that even in the absence of contraindications, MHCs were associated with a lower OAC prevalence.^15^ These findings are in line with the current work, where MHCs remained associated with a low rate of OAC initiation despite comprehensive adjustment for important bleeding risk factors.

The antithrombotic effects of several important psychotropic medications and the bleeding risks associated with them may be another factor influencing OAC prescribing in this population.^24^ Patients with MHCs often exhibit impaired self-care resources and medication adherence, which may provide additional concerns regarding the safe use of OAC medications.^25,26^ VKA anticoagulants necessitate a constant and disciplined lifestyle, continuous dose monitoring, and frequent dose modifications, which may prove tasking to some patients with MHCs. MHCs and cognitive difficulties sometimes associated with them might also negatively influence communication between patients and healthcare professionals, impairing shared decision-making. While often many of these concerns are justified, physicians may also at times be biased and harbour prejudice towards patients with MHCs when making treatment decisions.^27^ Furthermore, patients with MHCs may experience fragmented care due to the separation of psychiatric and somatic healthcare services and face barriers in access to care due to poor socioeconomic conditions, inadequate self-care resources, and other factors.^25^

Previous research has demonstrated that patients with MHCs receiving VKA anticoagulants have inferior OAC quality and experience more strokes and other adverse events compared with those without psychiatric comorbidity, although similar data from the NOAC era are all but lacking.^28–30^ The current findings are of clear clinical importance and add to the evidence that AF patients with MHCs constitute a large patient group who receive insufficient care for their condition.

There are some limitations regarding the current study that must still be acknowledged. The main limitation is the retrospective registry design of the study, which does not allow characterizing the study cohort as accurately as a prospective trial design, and so our findings are largely hypothesis generating in nature. MHCs were defined categorically from medical history, and condition severity and possible changes in mental health occurring during the study period could not be accounted for, which is a limitation. OAC initiation was determined by fulfilled prescriptions, and therefore, it is possible that in some cases patients who were prescribed OAC therapy never actually took their medication. We did not have data on the reasons for withholding OAC therapy in individual cases, which is an important limitation. Although our Poisson and Fine–Gray models were adjusted for baseline variables, the possibility of residual confounding may not be excluded. Recent data suggest that low income, low education status, and living alone are associated with a lower chance of OAC initiation among patients with AF.^31^ Indeed, a noted limitation of the current work is that we lacked data on socioeconomical factors, although due to the high reimbursement rates of medical treatment and full coverage of public health insurance in Finland these may not play a critical role in our results. Nevertheless, these data represent a large unselected nationwide cohort comprising all Finnish patients with incident AF during 2007–18 treated at any level of care, eliminating the risk of selection bias. A particular strength of the current study is the inclusion of primary care data as previous reports have mainly concentrated on secondary and tertiary care patients. Whether the observed differences in OAC initiation affect stroke outcomes in MHC patients ought to be determined in a future analysis.

In conclusion, MHCs are common among patients with newly diagnosed AF, and they are associated with a lower rate of OAC initiation compared with those without psychiatric comorbidity, even during the NOAC era.

## Supplementary Material

qcab077_Supplemental_File

## Data availability

The data underlying this article were provided by the Finnish Institute for Health and Welfare (THL), the Population Register Center, and the Social Insurance Institution (KELA) under license. Based on the contracts with the Finnish registries, restrictions apply to the availability of these data, and so they are not publicly available. Data are, however, available from the authors upon reasonable request and with permission of the Finnish Institute for Health and Welfare (THL), the Population Register Center, and the Social Insurance Institution (KELA)
