# Supplementary material for: The effect of mental health conditions on the use of oral anticoagulation therapy in patients with atrial fibrillation: the FinACAF study
Source: Eur Heart J Qual Care Clin Outcomes. 2021 Oct 22;8(3):269–76. doi: 10.1093/ehjqcco/qcab077 (PMC9071518; doi:10.1093/ehjqcco/qcab077)
Supplement: qcab077_Supplemental_File [file qcab077_supplemental_file.docx]

Supplementary material

**Table S1**. Definitions of the comorbidities

**Table S2.** Baseline characteristics of propensity score matched pairs

**Table S3.** The effect of MHCs on OAC initiation according to year of AF diagnosis

**eTable 1**. Definitions of the comorbidities

|  | ICD-10 | ICPC-2 | Reimbursement code | ATC code |
| --- | --- | --- | --- | --- |
| Hypertension | I10-I15 | K85, K86, K87 | 205 | C03A, C03B, C03DB, C03EA,  C07A, C08CA,  C08D, C09 |
| Dyslipidemia | E78 | T93 | 206 | C10 |
| History of heart failure | I50, I11.0, I13.0, I13.2 | K77 | 201 |  |
| Diabetes | E10-E14 | T89, T90 | 103, 215 | A10 |
| Previous stroke | I63, I64, I69.3-I69.8 | K90 |  |  |
| Vascular disease | I20-I25, I65-I66, I67.2, I70 | K74, K75, K76, K91, K92 | 206 |  |
| Bleeding history | D50.0, D62, D68.3, I60-I62, I69.0-I69.2, I85.0, I86.4, J94.2, K22.1, K22.3, K22.6, K25.0, K25.2,  K25.4, K25.6, K26.0, K26.2, K26.4, K26.6, K27.0, K27.2, K27.4, K27.6, K28.0, K28.2, K28.4, K28.6, K29.0, K62.5, K63.1, K63.3, K92.0-K92.2, N02, R04, R31, R58, S06.2-S06.6, S06.8 |  |  |  |
| Alcohol abuse | F10 |  |  |  |
| Renal failure or dialysis | N18, Z49 |  |  |  |
| Liver cirrhosis or failure | K70.2-K70.4, K71.7, K71.8, K72, K74 |  |  |  |
| Dementia | F00-F03, G30 |  |  |  |

Abbreviations: ATC, anatomic therapeutic chemical; ICD-10, International Classification of Diseases, Tenth Revision; ICPC-2, International Classification of Primary Care, Second Edition.

**eTable 2.** Baseline characteristics of propensity score matched pairs

|  | Unmatched pairs | | | Propensity score matched pairs | | |
| --- | --- | --- | --- | --- | --- | --- |
| Baseline characteristics | No MHC  n=191 675 | Any MHC  n=47 547 | Standardized differences | No MHC  n=46 095 | Any MHC  n=46 095 | Standardized differences |
| Age, years (95% CI) | 72.6 (72.6-72.7) | 72.8 (72.0-73.0) | 0.016 | 73.2 (73.0-73.3) | 72.9 (72.7-73.0) | 0.022 |
| Female sex | 90 754 (47.3) | 28 292 (59.5) | 0.246 | 28 224 (59.9) | 28 002 (59.5) | 0.010 |
| Hypertension | 147 803 (77.1) | 38 637 (81.3) | 0.102 | 38 421 (81.6) | 38 243 (81.2) | 0.010 |
| Dyslipidemia | 91 867 (47.9) | 23 854 (50.2) | 0.044 | 23 806 (50.5) | 23 584 (50.1) | 0.010 |
| History of heart failure | 31 886 (16.6) | 9 810 (20.6) | 0.103 | 9 541 (20.3) | 9 649 (20.5) | 0.006 |
| Diabetes | 40 143 (20.9) | 11 733 (24.7) | 0.089 | 11 456 (24.3) | 11 529 (24.5) | 0.004 |
| Previous stroke or TIA | 27 463 (14.3) | 8 605 (18.1) | 0.102 | 8 493 (18.0) | 8 444 (17.9) | 0.003 |
| Vascular disease^a^ | 48 815 (25.5) | 13 598 (28.6) | 0.070 | 13 546 (28.8) | 13 429 (28.5) | 0.006 |
| Renal failure or dialysis | 3 816 (2.0) | 1 197 (2.5) | 0.035 | 1 098 (2.3) | 1 173 (2.5) | 0.010 |
| Liver cirrhosis or failure | 911 (0.5) | 383 (0.8) | 0.041 | 310 (0.7) | 357 (0.8) | 0.012 |
| Alcohol abuse | 5 081 (2.7) | 4 354 (9.2) | 0.279 | 3 801 (8.1) | 3 930 (8.3) | 0.010 |
| Bleeding history | 20 625 (10.8) | 7 055 (14.8) | 0.122 | 6 951 (14.8) | 6 846 (14.5) | 0.006 |
| Dementia | 10 576 (5.5) | 7 335 (15.4) | 0.328 | 7 276 (15.4) | 6996 (14.9) | 0.017 |
| CHA_2_DS_2_-VASc score |  |  | 0.093 |  |  | 0.010 |
| 0 | 10 097 (5.3) | 1 796 (3.8) |  | 1 775 (3.8) | 1793 (3.8) |  |
| 1 | 21 137 (11.0) | 4 469 (9.4) |  | 4 320 (9.2) | 4 450 (9.4) |  |
| ≥2 | 160 441 (83.7) | 41 282 (86.8) |  | 41 000 (87.1) | 40 852 (86.7) |  |
| HAS-BLED score^b^ |  |  | 0.190 |  |  | 0.010 |
| 0 | 14 907 (7.8) | 2 560 (5.4) |  | 2 556 (5.4) | 2 560 (5.4) |  |
| 1 | 45 613 (23.8) | 9 985 (21.0) |  | 10 029 (21.3) | 9 968 (21.2) |  |
| 2 | 87 750 (45.8) | 20 578 (43.3) |  | 20 319 (43.1) | 20 521 (43.6) |  |
| ≥3 | 43 405 (22.6) | 14 424 (30.3) |  | 14 191 (30.1) | 14 046 (29.8) |  |
|  |  |  |  |  |  |  |

Values are presented as absolute number (percentage), mean (95% confidence interval) or median [interquartile range]

Abbreviations: CHA_2_DS_2_-VASc, congestive heart failure, hypertension, age ≥75 years, diabetes, history of stroke, vascular disease, age 65-74 years, sex category (female); CI, confidence interval; HAS-BLED, hypertension, abnormal renal function or liver enzymes, prior stroke, bleeding history, labile INR, elderly, drugs or alcohol; INR, international normalized ratio; MHC, mental health condition

^a^Coronary artery disease or peripheral vascular disease

^b^Modified HAS-BLED score without labile INR

Table S3. The effect of MHCs on OAC initiation according to year of AF diagnosis

| Year of AF diagnosis | Unadjusted SHR, 95% CI | Adjusted SHR, 95% CI | P value |
| --- | --- | --- | --- |
| 2007 | 0.763 (0.726-0.802) | 0.834 (0.793-0.876) | <0.001 |
| 2008 | 0.763 (0.727-0.800) | 0.836 (0.797-0.877) | <0.001 |
| 2009 | 0.751 (0.716-0.788 | 0.823 (0.784-0.864) | <0.001 |
| 2010 | 0.768 (0.732-0.805) | 0.850 (0.810-0.892) | <0.001 |
| 2011 | 0.772 (0.741-0.805) | 0.853 (0.818-0.890) | <0.001 |
| 2012 | 0.785 (0.754-0.818) | 0.858 (0.823-0.894) | <0.001 |
| 2013 | 0.773 (0.743-0.805) | 0.851 (0.818-0.887) | <0.001 |
| 2014 | 0.799 (0.768-0.833) | 0.891 (0.855-0.929) | <0.001 |
| 2015 | 0.822 (0.790-0.855) | 0.882 (0.846-0.919) | <0.001 |
| 2016 | 0.845 (0.813-0.879) | 0.908 (0.872-0.944) | <0.001 |
| 2017 | 0.852 (0.820-0.885) | 0.900 (0.866-0.936) | <0.001 |
| 2018 | 0.853 (0.819-0.887) | 0.911 (0.875-0.948) | <0.001 |

Risk estimates were adjusted for age, gender, hypertension, dyslipidemia, heart failure, diabetes, prior stroke or transient ischemic ischemia, vascular disease, renal failure or dialysis, liver cirrhosis or failure, alcohol abuse, prior bleeding episodes and dementia.

Abbreviations: AF, atrial fibrillation; CI, confidence interval; MHC, mental health condition; SHR, subdistribution hazard ratio
